# Perception of Dental Caries and Parental Difficulties in Implementing Oral Hygiene for Children Aged Less Than 6 Years: A Qualitative Study

**DOI:** 10.3390/dj8030062

**Published:** 2020-06-30

**Authors:** Marion Taormina, Sylvie Montal, Yoann Maitre, Paul Tramini, Estelle Moulis

**Affiliations:** 1 Department of Paedriatric Dentistry, Hospital and University of Montpellier, 34080 Montpellier, France; marionbentle@gmail.com (M.T.); estelle.moulis@umontpellier.fr (E.M.); 2 Department of Prosthodontics, Hospital and University of Montpellier, 34080 Montpellier, France; s-montal@chu-montpellier.fr; 3 Department of Dental Public Health, Hospital and University of Montpellier, 34080 Montpellier, France; maitreyoann@yahoo.fr

**Keywords:** early childhood caries, prevention, dental hygiene, qualitative methods, parental knowledge, parental difficulties

## Abstract

Background: Despite extensive prevention programs, dental hygiene remains inadequate, particularly among children under the age of six, and early childhood caries (ECC) are still a concern. Oral hygiene behavior and preventive practices seem difficult to change at a family level. Aim. The present study aimed to better understand the reasons behind this behavior and to identify the different barriers to the implementation of adequate preventive measures. Methods: A qualitative study was conducted in the pediatric dentistry service of the Montpellier University Hospital (France) in 2019. A thematic analysis concerning three domains was performed: family environment, dental literacy, and oral hygiene. Results. The main barriers encountered by the parents were, respectively, (1) a weakness in the organization of familial life, together with a low-medium family income and a lack of authority, (2) ignorance of the necessity of treating carious primary teeth, and (3) a lack of time for brushing or supervising their children’s teeth. Conclusion: These results showed that oral hygiene and primary teeth care could not be easily achieved in the family environment of the participants, and oral health strategies should be focused not only on children but also on their parents.

## 1. Introduction

Children’s oral health is an integral part of their overall health and influences their quality of life [1]. Regular public prevention programs and the involvement of pediatric dentists and dental surgeons have led to a decrease in caries incidence in France [2,3], but among specific at-risk populations, especially very young children, oral hygiene recommendations are not always complied with. Thus, a rise in the incidence of caries has been observed in the under six age group, referred to as early childhood caries (ECC). Many studies have shown that oral health is not equally distributed among social groups [4,5,6] and that there is a strong association between low socioeconomic status and ECC [7,8,9]. Thus, reducing caries incidence among children became the main concern in the priority education area.

ECC is defined as the presence of at least one decayed surface (with or without cavitation), a missing tooth (due to decay), or a filled tooth surface in any primary tooth in a child under the age of six [10].

ECC represents a major risk of caries in permanent teeth (adulthood) [11], and is sometimes associated with low birth weight or obesity [12]. The consequences of ECC are multiple: the impact on children’s quality of life, chronic pain, behavioral and sleep disorders [13,14], school absenteeism [15], and a lack of social integration [16]. The identification of risk indicators and the correction of modifiable risk factors are essential for the management of ECC. The first factor is microbial, with the early transmission of mutans *Streptococci* (MS) and *Lactobacilli* by the caregiver [17,18]. The second factor is poor dietary habits, linked to a high consumption of sugars at night (nursing bottle and/or breast-feeding ad libitum) and/or, during the day, sweet drinks and/or multi-daily snacks [19,20]. Enamel hypoplasia and a lack or ineffectiveness of toothbrushing (performed by the child alone) may also be risk factors, and reflect ECC multi-factorial aetiology [21].

Parents’ lack of knowledge and their inability to change family habits or to implement adequate toothbrushing techniques or children feeding contribute to maintaining a high level of ECC [22]. In view of this, it appeared interesting to evaluate the parents’ perception of carious disease and the main difficulties they encountered in implementing oral hygiene in children under the age of 6. To this purpose, a qualitative study was carried out in the pediatric dentistry department of the Montpellier University Hospital in 2019, using a semi-structured interview with parents of children under the age of 6. The secondary objective was to provide solutions to help parents change their family habits, and to achieve educational rules and behaviors leading to an improvement of their children’s oral health.

## 2. Materials and Methods

### Study Design and Sampling Strategy

This study follows the Standards for Reporting Qualitative Research (SRQR) [23]. In order to let parents openly express themselves on the topic of children’s oral health and preventive practices such as toothbrushing and dietary habits, individual semi-structured interviews were performed in a person-centered approach. Such an interview identifies topics to be addressed using open-ended questions and relaunch instructions. This approach is suitable to pinpoint concrete difficulties that patients may experience in daily practices. Each interview lasted between 30 and 60 min.

Individual interviews were conducted between January and March 2019, with a sample of parents recruited from the pediatric dentistry department of the Montpellier University Hospital (Centre de soins, d’enseignement et de recherche dentaire, Montpellier CHU, France). Parents were recruited sequentially until data saturation was obtained. All the parents who planned to attend the dental service were included on a voluntary basis during their child’s pediatric consultation. Thus, the parents were not selected nor identified before their first visit. They just had to be fluent in French to take part in this study. Their children had to be less than 6 years old, without any other restriction. The parents were interviewed in a quiet room at the first level of the dental service, while their children had dental treatments on the ground floor in the same service. Parents whose children were at low risk for caries were also included in order to obtain a higher number of verbatim responses regarding the different strategies and potential solutions.

During the interview, no advice was given because only the genuine information about the parents’ oral health knowledge was needed. Therefore, the parents had to express their daily difficulties in different domains, including in implementing their children’s oral hygiene.

**Figure 1:**
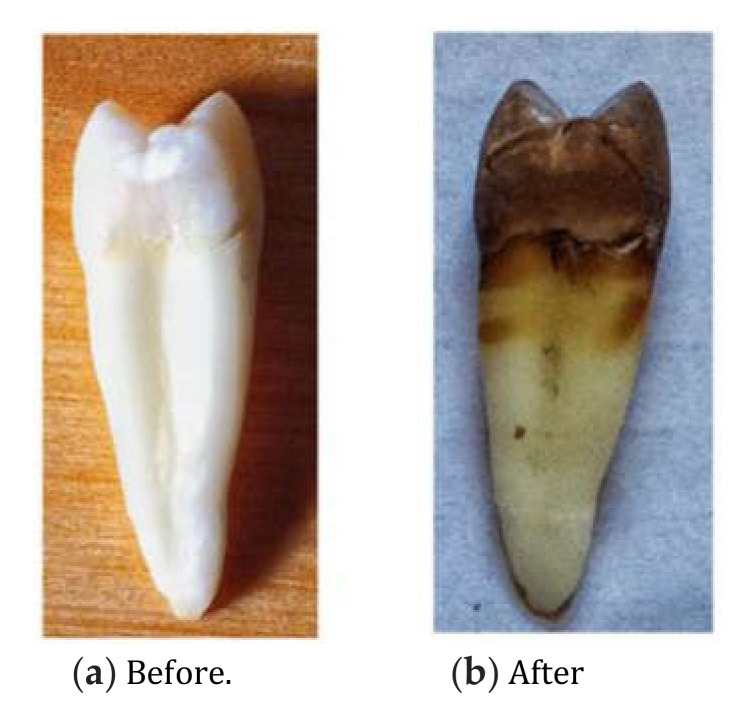
Color aspects of an extracted tooth before and after two weeks in a carbonated soft drink.

**Figure 2:**
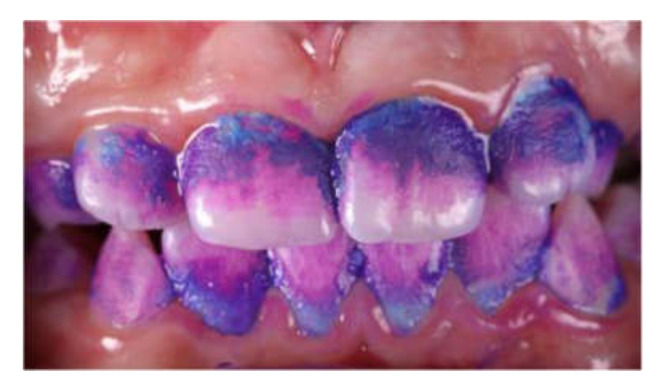
Dental plaque after application of a disclosing solution.

These interviews focused on the parents’ motivation, addressing three domains: family environment, dental literacy, and oral hygiene. Following the discussion on oral hygiene (third domain), two photographs relating to the same topic were presented to the parents. The first photograph (Figure 1) represented an extracted tooth before and after soaking it in a carbonated soft drink for two weeks, and the second (Figure 2) showed a child’s teeth that were covered with disclosing solution. These photographs helped to stimulate the responses in the interview concerning oral hygiene and nutrition.

## 3. Ethical Considerations

Participants were informed about the scope of this investigation and about the nature of the interview. Interviews were recorded with a dictaphone in order to be completely transcribed for analysis. Participants were also informed about the confidentiality and had the right to withdraw at any time from the study. Before each interview, participants had to provide written informed consent. Confidentiality of data (audio records and transcripts) was guaranteed, and the study was approved by the institutional ethics committee (Clinical Trials ID: RECHMPL18_0417, Date: 14 December 2018). It followed the principles of the Declaration of Helsinki and complied with international ethical standards.

### 3.1. Data Collection, Instruments, and Technology

One dentist (MT), with previous experience conducting qualitative studies, interviewed and collected all the data. She introduced herself to each participant in order to present the objectives, the rules, and the methods of the study, ensuring the interview was anonymous. The participants introduced themselves and provided information about their family situation, their occupation, and their social and economic background. The caries risk level was determined for each child. It was based on the American Association for Pediatric Dentistry recommendations for infants and children [24]. At the end of each interview, common recommendations on prevention along with the points that need to be improved were provided. The importance of their children’s caries risk level was discussed too, especially for their future dentition. Finally, solutions were proposed to the parents to help them to solve their difficulties in implementing oral hygiene. Individual interviews were conducted in a separate, quiet office of the pediatric dentistry department. The interviewer ensured that the discussion did not stay off topic and led it in a conversational tone, in a neutral, empathetic, and caring posture to create a climate of trust. All interviews were digitally audio-recorded, and later fully transcribed by the interviewer (MT). Data were collected until data saturation was reached, when no new information was obtained—“the point at which additional data do not improve understanding of the phenomenon under study” [25]. Therefore, the interviewer decided to stop including participants when parents reiterated what has been previously collected, or expressed the same beliefs or opinions. An interview guide that focused on parental difficulties and the possible solutions provided the basis for an in-depth discussion. The procedure was refined through the interviews, in order to be more efficient and to target the objective more precisely.

### 3.2. Data Processing and Analysis

A thematic analysis was performed, as described by Braun and Clark [26]. Thematic analysis is a widely used qualitative analytic method for identifying, analyzing, and reporting themes within a data corpus and interprets various aspects of the research topic [27]. The analysis comprised six steps:

1. Data transcription, reading and rereading data, and noting down initial ideas with the Word Microsoft^®^ software. This step involved another researcher from the pediatric department (EM);
2. Data coding: data were collected in literary form, so it was necessary to code interesting features of the data in a systematic form across the whole data set, and then to collate relevant data (MT and EM);
3. Searching for specific themes: clustering codes into potential topics and gathering all data relevant to each potential topic; two researchers (MT and EM) checked the credibility of the data in respect to the SRQR (audit trail and trustworthiness): the reliability between recorded interviews and the written text was double checked and their findings were compared;
4. Reviewing themes: modification, fusion of topics (when they were closely matching together), and/or the deletion of other topics, which was performed by the same researchers (MT and EM), who engaged in detailed discussions before making any decision; each interview was vertically analyzed (synthesis of the responses), then a horizontal reading was undertaken by theme (most significant responses by theme);
5. Theme description and illustration with data excerpts and citations;
6. Analysis report, interpretation, and discussion in relation to the study objectives; three researchers (MT, EM, and YM) took part in this discussion during several working sessions.

## 4. Results

Data saturation was obtained after interviewing 15 parents. Only one person (a father) refused to participate in the study, arguing that he was not interested. Another person (a mother) did not meet the selection criteria (language limitation). The participants confirmed that their children needed dental consultation, some of them just for a check-up visit. Table 1 summarizes the participants’ characteristics. Most of the parents (14/15) were married, and one third of the mothers were housewives and unemployed. Eighty percent of their children (12/15) showed a high caries risk level, and half of the children brushed their teeth daily. More than fifty percent (8/15) consumed soft drinks and had daily snacking habits.

### 4.1. First Part: Investigation of Three Domains

#### First Domain: Difficulties in Family Environment

This theme, describing the parents’ difficulties that they experienced in the family environment, showed a lack of authority and organization for the majority of the interviewed mothers. Participant 6: “Imposing rules is all well and good, but enforcing them is another story.” They often claimed that giving children a good education is harder nowadays. Participant 15: “If we impose rules, my kid feels frustrated and angry.” Participant 1: “I feel overwhelmed by work, home, and organization.” They had to repeatedly tell their children to brush their teeth, but children do not often heed the message. Parents admitted that, although their own oral hygiene practices were not adequate, their children exhibited a similar behavior. Participant 6 confessed that she never brushed her teeth, and her young child too. She also felt alone and had to deal with everything at home: “I’m the only one who takes care of children”; “I try to fulfil two roles, active woman and mom.” Participant 1: “I admit snacking every day, so I found it hard to prevent my own children from snacking.” The frequency of regular dental visits was far too insufficient, because parents claimed that they have not had enough time to take care of their own or their children’s oral health. Some of them waited too long and finally made an emergency dental visit (Participants 1, 4, 6, and 11). Parents had a poor image of the dentist, and unhappily, they transmitted it to their children. Therefore, they often postponed dental treatments, which also contributed to emergency dental visits. For some parents, dentists were associated with physical pain. Participant 1: “I’m anxious about dentist and dental treatments, so I avoid dental visits.” Participant 9: “I’m disappointed by dental treatments, because, despite daily oral hygiene and regular follow-ups, I have poor dentition and false teeth anyway.”

Another difficulty encountered by those families was giving up the evening bottle of milk before sleeping. Some parents claimed that physicians recommended it for their children. Participant 4: “Milk is good for child growth because an infant needs to increase his weight regularly.” Participant 7: “Milk is perfect for children and contains valuable antibodies and vitamins.” In the case of children that did not appreciate the taste of milk, their parents would add some sugar to it, or sometimes vanilla, chocolate, or honey (Participants 1, 3, and 6). They stated that, at the same time, children became comforted and quiet. Participant 8: “Taking his bottle of milk in bed stopped him from crying at night.” They knew that children are naturally inclined to sugar, sweets, soft drinks, and milk. It is a human basic need, and they found them very nice. Participant 3: “Sometimes, when he was a good child, he deserves a reward, like sweets or chocolate bars.” Some mothers said they bought sweets only for themselves, but they realized that those sweets were still within reach of their children (Participants 1, 11, and 14).

Parents found it hard to make appointments, because their dentist was very busy or the dental office was too far away (Participants 1 and 4). Therefore, visits to the dental office were very rare for these children.

Economically speaking, parents were ready to make financial efforts for their children’s health, including aesthetics and smiles (Participants 6, 7, and 8). However, they still had to go to the dental hospital, because dental treatments in private offices are too expensive. To compensate the time shortage, some parents proposed working part-time or enlisting childcare services (Participants 9 and 14).

### 4.2. Second Domain: Dental Literacy

Knowledge about toothbrushing practices was inadequate: the majority of the participants did not know the recommended technique, and some of them thought that horizontal toothbrushing was adequate (Participant 13) and that aggressive brushing was more efficient (Participant 10). Participant 5: “It’s unnecessary to start toothbrushing before 3 years of age.” Their knowledge about nutrition only concerned the negative impact of snacking and consuming sugar. However, they were not aware that many foods do contain sugar, such as starchy food, chips, fruits, or vegetables. They only knew about sweets, candies, ice cream, and pastries. Participants 6, 7, and 12 heard about the relationship between oral and systemic health, and were aware of the consequence of having good teeth beyond the oral cavity.

However, the main misconception was that primary teeth did not need to be treated. Participant 1: “Decayed milk teeth are not a problem, because they will fall out anyway.” Therefore, children wait too long before being treated, or even their parents never take care of their primary teeth. However, some mothers complained that they were misinformed about that (Participants 1, 2, and 10). All they knew was learned from the Internet, their parents, or their teachers at school, but not from health professionals (Participants 3, 5, 6, and 12). They thought that a motivational interviewing would be more efficient than a written recommendation because it is easier to remember (Participants 2 and 10). They do not want to read booklets (Participants 2 and 6).

### 4.3. Third Domain: Oral Hygiene

Some mothers claimed that their children often forgot to brush their teeth (Participants 1, 5, and 6), or sometimes played with their power or manual toothbrush rather than use it properly (Participants 3, 6, and 9). Participant 3: “Toothbrushing does not last long enough. My child salivates very much and forgets to brush his posterior teeth, because it makes him feel sick.” Moreover, some parents found that their dentist was not involved enough in teaching how to brush their children’s teeth (Participants 1, 2, 10, and 13). Some mothers recognized that their children did not brush their teeth properly, but they did not have the time to check with a plaque discloser (Participants 4, 10, and 14). Since they felt overwhelmed, parents often forgot to have their children brush their teeth after dinner. Participant 4: “Life is so stressing and in the evening time flies so fast; I forget even important things to do.” However, sometimes they just felt fed up with brushing every day (Participants 6 and 9). Some said that it would be more motivating to obtain a power brush and have good resolutions for daily toothbrushing. Participant 10: “I check the presence of dental plaque on my son’s teeth by scratching my nail on the surfaces of his teeth.” Participant 13 reported particular difficulties in implementing oral hygiene with her disabled child: “I have to wait until he decides it’s the best moment for him. Brushing his teeth really takes a long time; I have to sit him on a chair, wrap a towel around his neck, and use a power brush very carefully.” Most of the parents were impressed by the two photographs and became aware of the impact of soft drinks. Participant 6: “A plaque discloser would be used daily at home, because eyes can’t see everything.” Participant 8: “A single picture says more than many words.” Participant 5 suggested also considering the effect of sweets, ice creams, or chocolate bars on the teeth in Figure 1. However, Participant 4 stayed sceptical: “I have consumed soft drinks for a long time, but my teeth do not look like those of this picture” (referring to Figure 1), and Participant 15 had already seen this kind of picture and knew about the effects of soft drinks.

## 5. Discussion

These results show that oral hygiene and primary teeth care are not easily achieved in the family environment of the participants. The main difficulty for the parents in implementing good hygiene practices at home seems to be the motivation to change their own habits, and secondly, their oral health literacy [28]. The difficulty seems to be the transition from declarative knowledge to the use of that knowledge, which is often conceptualized as proceeding from awareness through acceptance to adoption [29]. Some authors showed that declarative and procedural knowledge are stored in separate areas within the brain (hippocampus and cortex, respectively) [30]. This concept can be applied for oral hygiene implementation: first, patients must be informed, and they then have to understand the information. The third step is to adapt these recommendations in daily practice, and health professionals can finally verify whether all of these measures have been efficiently implemented by patients. Other studies have demonstrated the influence of parents’ dental health habits on their children’s oral health [31,32,33,34]. They focused attention to the entire family, concerning their lifestyle and oral health habits.

The difficulty in enforcing rules was rather recurrent among parents, more particularly among mothers. Authority was considered as necessary, but at the same time, the parents recognized that they were weary: they took back instructions and eventually gave up on or ignored the problem. This attitude could be considered as permissive. The same result was found by Lotto et al. [35]: parental attitudes against the caries disease seemed to be inaccurate, influenced by their daily routines, doubts, and beliefs.

Transmission of negative emotions from parents to children was frequently found, but fear of the dentist did not appear predominant in the access to dental treatment. Knowledge about healthy feeding was quite unequally distributed, including the feeding bottle, but parents claimed that they were misinformed by their pediatrician. It seems that some pediatricians were not aware of all the preventive measures regarding ECC, as was reported in a Lebanese study [36].

Most of the interviewees complained about the lack of time after a hard day at work. Particularly, working mothers could not achieve adequate supervision of their children’s toothbrushing. Baiju et al. [37] found a significant association between employed mothers and higher dental caries experience among adolescents of Kerala (India). The same influence was found on general health: Shahraki et al. [38] found that mothers’ employment had a negative effect on children’s health at birth, but a mother’s education had a positive effect on children’s health at birth. These authors assume that mothers’ employment may have benefits for children, such as the purchase of healthy foods and entertainment tools, but it can reduce mothers’ ability to care for the child and decrease their time spent with the child. This seemed to be a constant finding in the family environment.

We have to underline the particular condition of this sample of patients attending the dental hospital. Most of them could be classified in a low-medium socioeconomic status and they chose the hospital for financial reasons. Therefore, the difficulties that they expressed in their environment could be specific to this sample of parents, but it also seemed more interesting because they are supposed to be more at risk for caries [28,39,40]. In France, “La Haute Autorité de Santé” (HAS) recommends focusing on preventing caries in single-parent families, taking into account a common risk approach [39]. It was most frequently a lone mother, who had to be both authoritarian and tolerant and did not have enough time to take care of her children. Some mothers, influenced by their daily routines and strong beliefs, appeared doubtful of specific diet recommendations, as found in a recent study [35].

Some parents complained that their dentist was too busy and not involved enough in teaching adequate toothbrushing. This study could highlight the leading role of dental hygienists, especially in a modern environment where general dental practitioners do not have enough time to give adequate recommendations about oral hygiene to all their patients. This is a sensitive issue particularly in France, where this occupation does not exist. Indeed, hygienists would spend enough time teaching proper brushing techniques and giving valuable information, especially to not neglect primary teeth, which are critically important for a child’s oral health.

### Limitations of the Study

The first limitation concerned the nature of the interviewer: It was a clinician who recruited sequentially the participants in the pediatric dentistry department of the hospital. This situation could influence a little the conduct of the interview, so we started creating a climate of trust. The interviewer intentionally did not wear a white coat and was talking in a simple way. She did not make any comment during the interviews about the participants’ behavior (“yes, good!”, or “it’s not a correct attitude”) and let them express freely. The patients attended the clinic for the first time, so there was no existing relationship between the interviewer and the participants. They were asked to talk frankly so that they could confess openly their difficulties.

Another limitation was to not use a focus group methodology; actually, it was difficult to put them into practice in the context of the hospital. We could have benefited from collective and individual responses provided by these focus groups. Nevertheless, oral hygiene counselling and tailored preventive measures are sometimes not adapted to this type of approach because difficulties encountered at home are so personal and do not suit everyone. The triangulation method could not be achieved in the data reviewing during the data analysing process. However, the credibility of the data was checked by the two researchers who were in charge of this process, with respect to the SRQR (audit trail and trustworthiness).

A lack of representability and a limited sample size could be addressed in this study, but in accordance with the SRQR guidelines for qualitative studies [23], a representative sample was not needed, and the sample size was determined on the data saturation. The purpose was to briefly highlight the parents’ profile and the problems they might encounter in implementing the oral hygiene of their children. However, further research needs to be conducted in order to obtain a wider panel of parent profiles and to better understand these difficulties. Follow-up studies would provide useful information on the efficiency of the solutions resulting from these interviews and on the persisting difficulties over time. Another drawback would be that the parents who agreed to participate in this study were probably the most motivated. It is a fact that reaching individuals at the highest risk of caries with the lowest motivation is a particularly sensitive issue.

One advantage of face-to-face interviews was that more spontaneous and sincere responses were obtained from parents, who felt more confident and well regarded by the interviewer. The interviewer had to be perceptive and create a climate of trust, without judgement. He had to use a neutral and empathetic posture to encourage parents to talk objectively and provide reliable answers—which are much more reliable than they are in public surveys that use conventional, auto-administered questionnaires. In our study, semi-structured interviews were supported by two photographs, which is rather unusual in qualitative research. Following the third domain of the interview, these photographs were meant to stimulate the participants’ comments on oral hygiene and nutrition. This alternative approach only occurred during the data collection process: the response treatment and data analysis were managed in the same way as the rest of the interview, through thematic analysis. It can be argued that these realistic photographs could influence the participants’ opinion about oral hygiene, or even make them feel guilty about their attitudes. Indeed, these photographs provided more concrete evidence than any concept addressed in the interviews. However, we noticed that instead of portraying signs of guilt, observing the photographs stimulated the parents and got them talking in an uninhibited manner. Therefore, the use of photographs seemed to be an effective tool in the data collection process.

## 6. Conclusions

This qualitative study allowed us to better understand the family environment; in particular, parents were aware that most young children tended to replicate their parents’ behavior, instead of following good recommendations. The first conclusion would be to encourage parents and caregivers to change their own oral hygiene practices, in the presence of their children, if that is possible. It appeared that parents’ oral health attitudes may have a direct influence on the caries experience of their children, indicating that oral health strategies should be focused not only on children but also on their parents. The second conclusion would be to inform physicians and pediatricians about the harmful impact of milk just before sleeping; in this way, cooperation between the dentist and the physician could be improved. As a final point, the parents stay in the center of their decisions: more authority and responsibility, which may allow the next step of patient care to be achieved: educational therapy.

## Figures and Tables

**Table 1:** Description of participants’ characteristics.

Number	Parent	Parental Status	Children and Age	Occupation	Caries Risk Level
1	Mother	Married	3: 4, 9, and 12 years	Yes Nursing auxiliary	High
2	Mother	Married	3: 4, 6, and 8 years	No Housewife	High
3	Mother	Married	3: 1, 4, and 6 years	Yes Commercial employee (parental leave)	High
4	Father	Married	2: 2 months and 3 years	Yes Branch manager	High
5	Grand-mother	Parents married	2: 6 years twins	Non retired Yes for the parents	High
6	Mother	Single parent	2: 5 and 6 years	No Housewife	High
7	Mother	Married	3: 3, 6, and 8 years	No Housewife	High
8	Father	Married	3: 4, 6, and 11 years	Yes Mason	High
9	Mother	Married	2: 6 and 9 years	Yes Assistant nurse	High
10	Mother	Married	2: 6 and 10 years	Yes Commercial	High
11	Mother	Married	5: 5, 9, 11, 13, and 14 years	Yes Company manager	High
12	Father	Married	1: 3 years	Yes Self-contractor	Low
13	Mother	Married	2: 6 and 12 years	Yes Self-contractor	High
14	Mother	Married	1: 5 years	Yes Admin assistant	Low
15	Mother	Married	1: 3 years	Yes Pediatrician	Low
